# Supplementary material for: Changes in ferrous iron and glutathione promote ferroptosis and frailty in aging Caenorhabditis elegans
Source: eLife. 2020 Jul 21;9:e56580. doi: 10.7554/eLife.56580 (PMC7373428; doi:10.7554/eLife.56580)
Supplement: Supplementary file 1. [file elife-56580-supp1.docx]

**Glutathione and aging**

There was a significant reduction in glutathione levels with increased adult age in *C. elegans* (**Figure 1c**). For comparisons between age and treatment groups an Ordinary one-way ANOVA was performed, followed by Tukey’s multiple comparisons test (ANOVA: F (3, 20) = 32.96, *p*< 0.0001). The results of the pairwise comparisons, corrected for multiple comparisons, are shown in **Table 1**.

**Table 1:** Summary of glutathione level comparisons between ages

| Tukey's multiple comparisons test | Mean Diff. | 95.00% CI of diff. | Significant? | Adjusted *p* Value |
| --- | --- | --- | --- | --- |
| Day 1 vs. Day 4 | 0.1964 | 0.08439 to 0.3085 | Yes | 0.0003 |
| Day1 vs. Day 8 | 0.2738 | 0.1618 to 0.3858 | Yes | <0.0001 |
| Day 1 vs. Day 10 | 0.3667 | 0.2546 to 0.4787 | Yes | <0.0001 |
| Day 4 vs. Day 8 | 0.07738 | -0.03466 to 0.1894 | No | 0.2992 |
| Day 4 vs. Day 10 | 0.1702 | 0.0582 to 0.2823 | Yes | 0.0015 |
| Day 8 vs. Day 10 | 0.09286 | -0.01918 to 0.2049 | No | 0.1423 |

**Glutathione depletion**

There was a significant reduction in glutathione levels after treatment with of 4 Day old adults with DEM (**Figure 1f**)*.* Pre-treatment with SIH protected against this reduction, with this treatment also resulting in a higher basal level of glutathione. For comparisons between treatment groups an Ordinary one-way ANOVA was performed, followed by Tukey’s multiple comparisons test (ANOVA: F (5, 30) = 50.97, *p*< 0.0001). The results of the pairwise comparisons, corrected for multiple comparisons, are shown in **Table 2.**

**Table 2:** Summary of glutathione level comparisons after DEM exposure with pre-treatment

| Tukey's multiple comparisons test | Mean Diff. | 95 % CI of diff. | Significant? | Adjusted *p* Value |
| --- | --- | --- | --- | --- |
| Day 4 Control vs Day 4 Lip-1 | -0.04508 | -0.3588 to 0.2686 | No | 0.9997 |
| Day 4 Control vs Day 4 SIH | -0.6447 | -0.9584 to -0.331 | Yes | <0.0001 |
| Day 4 Ctl vs. Day 4 Ctl + DEM | 0.6921 | 0.409 to 0.9752 | Yes | <0.0001 |
| Day 4 Lip-1 vs. Day 4 Lip-1 + DEM | 0.7379 | 0.4515 to 1.024 | Yes | <0.0001 |
| Day 4 SIH vs. Day 4 SIH + DEM | 0.7667 | 0.4857 to 1.048 | Yes | <0.0001 |
| Day 4 Ctl + DEM vs. Day 4 Lip-1 + DEM | 0.0006989 | -0.2518 to 0.2532 | No | >0.9999 |
| Day 4 Ctl + DEM vs. Day 4 Lip-1 + DEM | -0.5701 | -0.8166 to -0.3237 | Yes | <0.0001 |
