# Supplementary material for: Changes in ferrous iron and glutathione promote ferroptosis and frailty in aging Caenorhabditis elegans
Source: eLife. 2020 Jul 21;9:e56580. doi: 10.7554/eLife.56580 (PMC7373428; doi:10.7554/eLife.56580)
Supplement: Supplementary file 2. [file elife-56580-supp2.docx]

**Analysis of Iron from X-ray Fluorescence Microscopy**

**Total Mean areal density of iron analysis**

Summary statistics and tests for normality of areal density for iron (pg µm^-2^) are included in **Table 1**. All the total iron data sets were normally distributed, as indicated below.

**Table 1**: Summary of areal density iron results between treatments and ages

|  | Day 1 | Day 4 Control | Day 4  SIH | Day 4  Lip-1 | Day 8 Control | Day 8  SIH | Day 8  Lip-1 |
| --- | --- | --- | --- | --- | --- | --- | --- |
| Number of values | 32 | 25 | 27 | 20 | 12 | 17 | 22 |
|  |  |  |  |  |  |  |  |
| Minimum | 177.7 | 443.4 | 267.1 | 590.5 | 629.6 | 324.2 | 644 |
| 25% Percentile | 216.2 | 592.2 | 298.3 | 630.3 | 677.6 | 381.9 | 726.5 |
| Median | 236 | 663.6 | 304.4 | 711.4 | 823.8 | 408.9 | 800.5 |
| 75% Percentile | 254.9 | 739.1 | 357.3 | 732.2 | 885.3 | 501.9 | 892.4 |
| Maximum | 271.7 | 894 | 412.1 | 851.8 | 1100 | 558 | 1143 |
|  |  |  |  |  |  |  |  |
| Mean | 234 | 666.2 | 321.8 | 698.5 | 820.3 | 434.7 | 821.6 |
| Std. Deviation | 26.61 | 111.3 | 37.49 | 67.26 | 145.1 | 74.49 | 137.4 |
| Std. Error of Mean | 4.704 | 22.26 | 7.216 | 15.04 | 41.87 | 18.07 | 29.29 |
|  |  |  |  |  |  |  |  |
| Lower 95% CI of mean | 224.4 | 620.3 | 307 | 667 | 728.2 | 396.4 | 760.7 |
| Upper 95% CI of mean | 243.6 | 712.1 | 336.7 | 730 | 912.5 | 473 | 882.5 |
| D'Agostino & Pearson normality test | | | | | | | |
| K2 | 2.672 | 0.2089 | 3.471 | 0.4574 | 0.6834 | 1.74 | 5.565 |
| *p* value | 0.2629 | 0.9008 | 0.1763 | 0.7956 | 0.7105 | 0.4190 | 0.0619 |
| Passed normality test (α=0.05)? | Yes | Yes | Yes | Yes | Yes | Yes | Yes |
| *p* value summary | ns | ns | ns | ns | ns | ns | ns |

There was a significant difference between mean areal density of iron (F (6, 148) = 171.3, *p* < 0.0001) amongst the groups measured. Comparisons between age and treatment groups an Ordinary one-way ANOVA was performed, followed by Sidak’s multiple comparisons test. The results of the pairwise comparisons, corrected for multiple comparisons, are shown in **Table 2**.

**Table 2:** Summary of areal density of iron comparisons between ages and treatments

| Sidak's multiple comparisons test | Mean Diff. | 95.00% CI of diff. | Significant? | Adjusted *p* value |
| --- | --- | --- | --- | --- |
| Day 1 Control vs. Day 4 Control | -432.2 | -499.3 to -365.1 | Yes | <0.0001 |
| Day 1 Control vs. Day 8 Control | -586.3 | -671.5 to -501.2 | Yes | <0.0001 |
| Day 4 Control vs. Day 8 Control | -154.1 | -242.4 to -65.85 | Yes | <0.0001 |
| Day 4 Control vs. Day 4 SIH | 344.4 | 274.6 to 414.1 | Yes | <0.0001 |
| Day 4 Control vs. Day 4 Lip-1 | -32.28 | -107.7 to 43.15 | No | 0.9226 |
| Day 8 Control vs. Day 8 SIH | 385.6 | 290.9 to 480.4 | Yes | <0.0001 |
| Day 8 Control vs. Day 8 Lip-1 | -1.229 | -91.45 to 89 | No | >0.9999 |
| Day 4 SIH vs. Day 8 SIH | -112.9 | -190.7 to -35.02 | Yes | 0.0006 |
| Day 4 Lip-1 vs. Day 8 Lip-1 | -123.1 | -200.8 to -45.42 | Yes | 0.0001 |
| Day 4 SIH vs. Day 4 Lip-1 | -376.6 | -450.8 to -302.5 | Yes | <0.0001 |
| Day 8 SIH vs. Day 8 Lip-1 | -389.9 | -469 to -304.8 | Yes | <0.0001 |

**Total body iron analysis**

Summary statistics and tests for normality of total body iron (pg) are included in **Table 3**. All the total iron data sets were normally distributed, as indicated below.

**Table 3**: Summary of total body iron results between treatments and ages

|  | Day 1 | Day 4 Control | Day 4  SIH | Day 4  Lip-1 | Day 8 Control | Day 8  SIH | Day 8  Lip-1 |
| --- | --- | --- | --- | --- | --- | --- | --- |
| Number of values | 32 | 25 | 27 | 20 | 12 | 17 | 22 |
|  |  |  |  |  |  |  |  |
| Minimum | 13.48 | 91.73 | 41.07 | 71.51 | 122.8 | 74.16 | 100 |
| 25% Percentile | 22.06 | 109.2 | 61.28 | 91.34 | 134.3 | 117.5 | 128.9 |
| Median | 25.36 | 124.4 | 66.78 | 114.6 | 146.6 | 127.5 | 157.2 |
| 75% Percentile | 27.75 | 138.6 | 71.8 | 129.5 | 173.2 | 169.5 | 189.6 |
| Maximum | 39.6 | 171.1 | 98.92 | 148.6 | 214.2 | 202 | 277.9 |
|  |  |  |  |  |  |  |  |
| Mean | 24.79 | 126.1 | 68.37 | 113.3 | 154.4 | 138.6 | 165.1 |
| Std. Deviation | 5.166 | 21.05 | 12.1 | 21.91 | 27.94 | 36.58 | 44.93 |
| Std. Error of Mean | 0.9133 | 4.211 | 2.328 | 4.9 | 8.065 | 8.873 | 9.579 |
|  |  |  |  |  |  |  |  |
| Lower 95% CI of mean | 22.93 | 117.4 | 63.59 | 103.1 | 136.7 | 119.8 | 145.2 |
| Upper 95% CI of mean | 26.65 | 134.8 | 73.16 | 123.6 | 172.2 | 157.4 | 185 |
| D'Agostino & Pearson normality test | | | | | | | |
| K2 | 2.708 | 0.8963 | 4.252 | 1.75 | 3.123 | 0.6397 | 4.128 |
| *p* value | 0.2582 | 0.6388 | 0.1193 | 0.4169 | 0.2099 | 0.7262 | 0.1270 |
| Passed normality test (α=0.05)? | Yes | Yes | Yes | Yes | Yes | Yes | Yes |
| *p* value summary | ns | ns | ns | ns | ns | ns | ns |

There was a significant difference between total body iron (F(6,148)=97.3, *p* < 0.0001) amongst the groups measured. Comparisons between age and treatment groups an Ordinary one-way ANOVA was performed, followed by Sidak’s multiple comparisons test. The results of the pairwise comparisons, corrected for multiple comparisons, are shown in **Table 4**.

**Table 4:** Summary of total body iron between ages and treatments.

| Sidak's multiple comparisons test | Mean Diff. | 95.00% CI of diff. | Significant? | Adjusted *p* Value |
| --- | --- | --- | --- | --- |
| Day 1 Control vs. Day 4 Control | -101.3 | -120.9 to -81.67 | Yes | <0.0001 |
| Day 1 Control vs. Day 8 Control | -129.6 | -154.5 to -104.8 | Yes | <0.0001 |
| Day 4 Control vs. Day 8 Control | -28.35 | -54.16 to -2.538 | Yes | 0.0211 |
| Day 4 Control vs. Day 4 SIH | 57.71 | 37.31 to 78.11 | Yes | <0.0001 |
| Day 4 Control vs. Day 4 Lip-1 | 12.74 | -9.306 to 34.79 | No | 0.6818 |
| Day 8 Control vs. Day 8 SIH | 15.8 | -11.91 to 43.51 | No | 0.6991 |
| Day 8 Control vs. Day 8 Lip-1 | -10.7 | -37.07 to 15.68 | No | 0.9550 |
| Day 4 SIH vs. Day 8 SIH | -70.26 | -93.01 to -47.5 | Yes | <0.0001 |
| Day 4 Lip-1 vs. Day 8 Lip-1 | -51.79 | -74.49 to -29.08 | Yes | <0.0001 |
| Day 4 SIH vs. Day 4 Lip-1 | -44.97 | -66.65 to -23.29 | Yes | <0.0001 |
| Day 8 SIH vs. Day 8 Lip-1 | -26.49 | -50.23 to -2.763 | Yes | 0.0178 |
