# Supplementary material for: Changes in ferrous iron and glutathione promote ferroptosis and frailty in aging Caenorhabditis elegans
Source: eLife. 2020 Jul 21;9:e56580. doi: 10.7554/eLife.56580 (PMC7373428; doi:10.7554/eLife.56580)
Supplement: Supplementary file 3. [file elife-56580-supp3.docx]

**Estimation of Fe^2+^**

There was a significant difference between the fractional Fe^2+^/Fe(total) estimates, determined by non-overlapping 95% CI, between aged TJ1060 animals. Treatment with Lip-1 or SIH restored the Fe^2+^/Fe(total) estimate. Similarly, treatment of wild type (N2) animals with DEM markedly increased Fe^2+^/Fe(total).

**Table:** Summary of the estimated Fe^2+^/Fe(total) for each treatment group.

|  | Group | Mean Fe^2+^/Fe(total) | 95% CI |
| --- | --- | --- | --- |
| **TJ1060** | Day 1 | 0.239 | 0.233-0.244 |
|  | Day 8 Control | 0.300 | 0.295-0.305 |
|  | Day 8 Lip-1 | 0.231 | 0.198-0.262 |
|  | Day 8 SIH | 0.236 | 0.232-0.240 |
|  |  |  |  |
| **N2** | Day 4 | 0.228 | 0.223-0.233 |
|  | Day 4 + DEM | 0.310 | 0.305-0.314 |
