# Supplementary material for: Changes in ferrous iron and glutathione promote ferroptosis and frailty in aging Caenorhabditis elegans
Source: eLife. 2020 Jul 21;9:e56580. doi: 10.7554/eLife.56580 (PMC7373428; doi:10.7554/eLife.56580)
Supplement: Supplementary file 4. [file elife-56580-supp4.docx]

**Lifespan analysis**

Summary of survival data from 8 **independent** replicate experiments. Median and maximum lifespan figures are days of adulthood at 25 (±1) °C. Censored individuals are those that were lost, primarily due to crawling off the side of the plate. Median lifespan was initially compared using a Log-rank (Mantel-Cox) test. * p<0.0001; **†** p=0.0013

|  | **control** | | | | **250 μM SIH** | | | | | **200 μM Lip-1** | | | | |
| --- | --- | --- | --- | --- | --- | --- | --- | --- | --- | --- | --- | --- | --- | --- |
| Replicate | death events | censored | **median** | **max** | death events | censored | **median** | max | % median ⇑ | death events | censored | **median** | max. | % median ⇑ |
| 1 | 88 | 3 | **7** | 17 | 71 | 4 | **14^*^** | 19 | 100 | 103 | 1 | **13^*^** | 25 | 86 |
| 2 | 61 | 10 | **8** | 16 | 96 | 7 | **14^*^** | 19 | 75 | 88 | 6 | **11^†^** | 20 | 38 |
| 3 | 109 | 3 | **9** | 21 | 81 | 7 | **16^*^** | 23 | 78 | 108 | 2 | **14^*^** | 24 | 56 |
| 4 | 99 | 1 | **7** | 19 | 111 | 10 | **16^*^** | 20 | 129 | 147 | 1 | **14^*^** | 24 | 100 |
| 5 | 91 | 16 | **9** | 19 | 93 | 9 | **14^*^** | 18 | 56 | 86 | 7 | **14^*^** | 26 | 56 |
| 6 | 97 | 11 | **8** | 16 | 105 | 10 | **14^*^** | 24 | 75 | 112 | 6 | **12^*^** | 25 | 50 |
| 7 | 93 | 2 | **7** | 21 | 90 | 29 | **17^*^** | 29 | 143 | 80 | 6 | **15^*^** | 27 | 114 |
| 8 | 72 | 5 | **8** | 22 | 73 | 13 | **20^*^** | 26 | 150 | 85 | 5 | **14^*^** | 26 | 75 |
|  |  |  |  |  |  |  |  |  |  |  |  |  |  |  |
| Total | 709 | 51 |  |  | 720 | 89 |  |  |  | 809 | 34 |  |  |  |
| **Mean** |  |  | **8** | **19** |  |  | **16** | **22** | **+101%** |  |  | **13** | **25** | **+72%** |
