# Supplementary material for: Changes in ferrous iron and glutathione promote ferroptosis and frailty in aging Caenorhabditis elegans
Source: eLife. 2020 Jul 21;9:e56580. doi: 10.7554/eLife.56580 (PMC7373428; doi:10.7554/eLife.56580)
Supplement: Supplementary file 5. [file elife-56580-supp5.docx]

**Testing Departure from Temporal Rescaling**

Following the recently published results of Stroustrup *et al*. (Stroustrup et al., 2016) we determined whether the results observed with both the SIH and Liproxstatin interventions were due to temporal scaling of aging.

Using the modified Kolmogorov-Smirnov (K-S) test (Fleming et al., 1980) we examined whether the treatment effects can be reasonably modelled using the Accelerated Failure Time (AFT) model to determine whether we can reasonably assume that the treatment effect manifests in temporal rescaling. To control for inter-replicate differences, the **test is conducted on the residuals a replicate-specific AFT model with the Buckley-James method** (Buckley and James, 1979) **using the nonparametric baseline hazards form**. The function *bj* in R package *rms* was used to fit the models. The null hypothesis for the two-sample K-S test is that the simple temporal rescaling holds and the residuals for the two treatment groups under comparison come from the same distribution.

Using the modified Kolmogorov-Smirnov (K-S) test, we examined whether the treatment effects can be reasonably modelled using the AFT model to determine whether we can reasonably assume that the treatment effect manifests in temporal rescaling. To control for inter-replicate differences, the **test is conducted on the residuals from replicate-specific AFT model with the Buckley-James method** **that uses nonparametric baseline hazards form**. The function *bj* in R package *rms* was used to fit the models. The null hypothesis for the two-sample K-S test is that the simple temporal rescaling holds and the residuals for the two treatment groups under comparison come from the same distribution.

Since the R function can only take right-censored data and our lifespan data are interval-censored, we use the mid-point of the interval to assign the time of event. Treating interval-censored data as right-censored is expected to underestimate the variability in the statistical estimates (Lindsey and Ryan, 1998) which in turn will produce an optimistic (smaller than it should be) *p*-value. To reduce the likelihood of false rejection of the null hypothesis merely because of the optimistic *p*-value, we chose a more stringent Type I error (0.01) than the usual 0.05 when conducting the K-S test.

**Table 1**: *p*-values of KS test on Residuals of nonparametric AFT models

| Replicate | Control *vs* Lip-1 | Control *vs* SIH | Lip-1 *vs* SIH |
| --- | --- | --- | --- |
| 1 | 9×10^-5^ | 2×10^-3^ | 1×10^-15^ |
| 2 | 2×10^-2^ | 4×10^-3^ | 8×10^-5^ |
| 3 | 7×10^-3^ | 9×10^-4^ | 4×10^-11^ |
| 4 | 4×10^-13^ | 3×10^-15^ | 2×10^-31^ |
| 5 | 2×10^-4^ | 1×10^-4^ | 7×10^-28^ |
| 6 | 1×10^-9^ | 3×10^-5^ | 2×10^-5^ |
| 7 | 3×10^-6^ | 4×10^-14^ | 1×10^-7^ |
| 8 | 1×10^-6^ | 4×10^-6^ | 1×10^-8^ |

As can be seen from **Table 1**, the effect of Lip-1 and SIH treatment relative to control always deviates away from simple temporal rescaling (all *p*-values < 10^-2^) with the exception of Lip-1 in replicate 2. **Figure 1** shows graphically why the AFT assumption is not reasonable since the survival curves of residuals from the AFT models show ‘crossing’ behavior. If the simple temporal rescaling assumption is reasonable, we would expect the survival curves for the different treatments to be very similar to each other. The observed crossing of the curves is primarily caused by the de-acceleration in the survival function for control worms.

When all the replicates are combined, and the modified KS test were performed on the residuals of the AFT models with the best parametric form, we found that the *p*-value for comparing Control *vs* Lip-1, Control *vs* SIH and Lip-1 *vs* SIH are 2 x 10^-24^, 1 x 10^-24^ and 3 x 10^-37^ respectively. These results indicate that failure to control for inter-replicate differences would lead to even stronger evidence of departure from simple temporal rescaling.


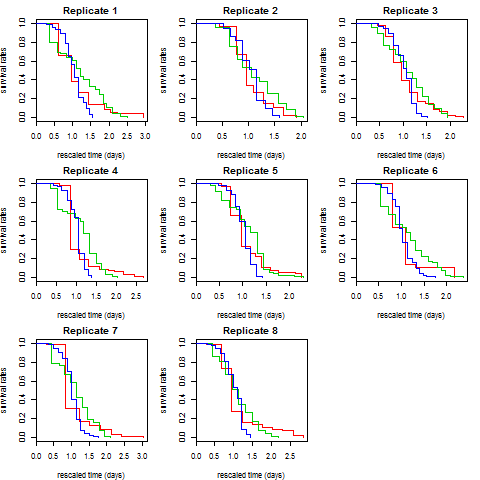


**Figure 1**: The residuals of AFT model with nonparametric hazards form for different replicates (red = control, green = Lip-1, blue = SIH). The x-axis has been rescaled to remove the temporal rescaling effect. Under the null hypothesis, we expect the residuals for the different groups to have the same survival curves.

**Determining AFT models with the best baseline hazard form**

In order to investigate the possible reasons for departure from simple temporal rescaling, we used parametric survival models which require specification of a parametric baseline hazard form. To minimize the risk of model misspecification, we identified the most appropriate baseline hazard form for each replicate using the Bayesian Information Criterion (BIC), with the best parametric form chosen as the model that minimizes the BIC.

The following parametric baseline hazards were fitted:Gompertz, Gompertz with Frailty, Weibull, Weibull with Frailty, Log-normal and Log-logistic. The mathematical formulae for each parametric form are detailed below:

Gompertz: h(t|a,b) = (a/b)exp(t/b )

Gompertz with frailty: h(t|a,b,σ) = (a/b)exp(t/b)/[1 + σ^2^a exp((t/b) − 1 )]

Weibull: h(t|α,β) = (α/β)(t/β)^α−1^

Weibull with frailty: h(t|α,β,σ) = (α/)(t/β)^α−1^/[1 + σ^2^(t/β)^α^]

Log-normal: h(t|µ,σ) = ϕ((log t − µ)/σ)/σt[1 − Φ ((log t − µ)/σ)]

Log-logistic: h(t|α,β) = (α/β)(t/β)^α−1^[1 + (t/β)^α^]

Here *ϕ* and Φ denote the probability density function (PDF) and cumulative distribution function (CDF), respectively, of the standard normal distribution; *µ* and *σ* denote the mean and standard deviation (in the case of the log-normal, the mean and standard deviation of the logarithm of *x*); *λ*, *α*, and *a* are shape parameters; *β* and *b* are scale parameters. In the case of frailty, individual hazards *hi*(*t*) are related to a baseline hazard by a random factor *Z* that follows a Gamma distribution with mean 1 and variance *σ*2.

Bayesian Information Criterion (BIC) is used to determine the best parametric form of the hazards; with better fit indicated by lower BIC value. All computations are done using flexsurv R package, taking into account that events are interval censored to account for the fact that we do not observe the exact event time and only know that events occurred within an interval (a,b).


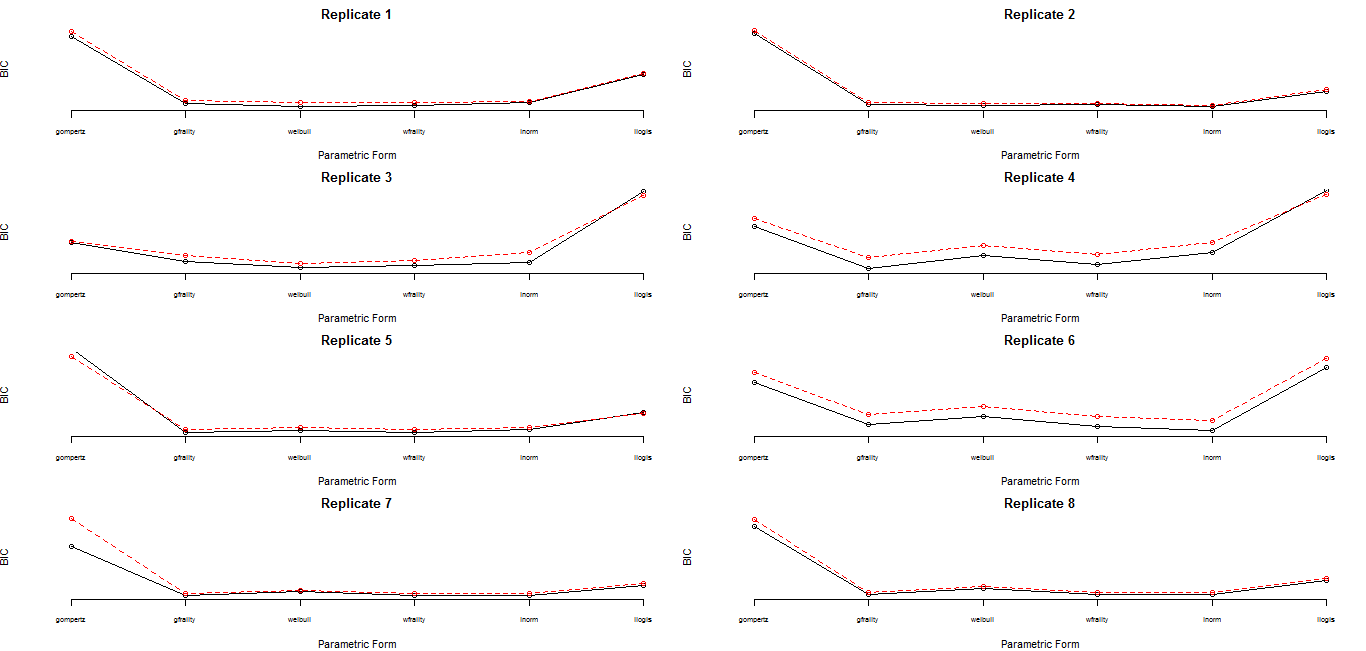


**Figure 2**: Bayesian Information Criterion (BIC) for AFT models with different parametric baseline hazard forms

From **Figure 2**, we can see that Gompertz baseline hazard form does not fit the data well, except when frailty is used. Weibull baseline hazard fits some replicates quite well and the fit is further improved when frailty is assumed. In fact, Weibull with frailty provides the best parametric baseline hazards form for nearly all the replicates, followed closely by the log-normal models.

**Possible Causes of Departure from Temporal Rescaling**

Stroustrup *et al.* (Stroustrup et al., 2016) show that unobserved heterogeneity (*e.g.* due to heterogeneity in the temperature the worms were exposed to) could cause de-acceleration and further, when the degree of heterogeneity is different between treatments, this could give rise to apparent departure from temporal rescaling. We investigated whether there is significant difference in the degree of heterogeneity by comparing two models for each replicate: **(M1)** model with Weibull frailty (Weibull hazard, Gamma frailty) where the degree of heterogeneity (represented by parameter σ^2^ and *a*) is assumed to be the same for all three treatments, **(M2)** where the parameter σ^2^ is allowed to be different but parameter *a* fixed across treatments and **(M3)** where the parameter σ^2^ and a *are* allowed to be different across treatments. We compared the three models based on their BIC values and also performed likelihood ratio tests, comparing M1 *vs* M2 and M1 *vs* M3.

**Note that only M1 can be classified as an AFT model while M2 and M3 are not AFT models**, as the treatment effects also manifest in the other parameters apart from the location (shift) parameter. **Table 2** shows that both M3 and M2 provide better fit than M1 for all replicates as indicated by small likelihood ratio test (LRT) p-values, with M3 providing more convincing p-values.

**Table 2:** BIC values for AFT model with Weibull frailty baseline hazards (M1), non-AFT model with Weibull frailty baseline hazards and treatment-dependent heterogeneity levels σ^2^(M2) and non-AFT model with Weibull frailty baseline hazards and treatment-dependent shape parameter (a) and heterogeneity levels σ^2^(M3)

| Replicate | M1 | M2 | M3 | LRT P-value  (M2 *vs* M1) | LRT P-value  (M3 *vs* M1) |
| --- | --- | --- | --- | --- | --- |
| 1 | 1325.3 | 1321.0 | 1289.4 | 2×10^-4^ | 2×10^-12^ |
| 2 | 1090.3 | 1080.7 | 1081.8 | 1×10^-5^ | 1×10^-6^ |
| 3 | 1503.6 | 1495.5 | 1476.0 | 3×10^-5^ | 8×10^-11^ |
| 4 | 1613.3 | 1590.7 | 1500.0 | 1×10^-8^ | 0 |
| 5 | 1263.5 | 1246.4 | 1224.0 | 3×10^-7^ | 2×10^-13^ |
| 6 | 1464.3 | 1431.8 | 1408.2 | 8×10^-11^ | 0 |
| 7 | 1079.0 | 1066.4 | 1034.1 | 3×10^-6^ | 1×10^-14^ |
| 8 | 1033.3 | 1016.0 | 1004.0 | 3×10^-7^ | 5×10^-11^ |

The fitted survival curves for M3 model in each replicate are compared to the observed curves in **Figure 3**. To investigate whether M3 provides an adequate fit to the data, for each replicate, we performed a chi-square goodness of fit test, comparing the observed survival curve to the fitted curve for each treatment group. The results are presented in **Table 3**. While the controls and SIH are always well-fitted by the Weibull frailty models (all *p*-values >0.01), the Lip-1 data from replicates 1, 4, 5 and 7 are not adequately fitted by the Weibull frailty model. The lack of fit for replicate 4 in particular is mainly caused by the estimated survival underestimating the observed counterparts in the middle-section between 7 and 15 days and overestimation on the tails.

**Table 3:** Chi-square Goodness of Fit *p*-value for M3 (non-AFT model with treatment-dependent shape and heterogeneity parameters)

| Replicate | Control | Lip-1 | SIH |
| --- | --- | --- | --- |
| 1 | 0.4 | 4×10^-4^ | 0.8 |
| 2 | 0.08 | 0.07 | 0.1 |
| 3 | 0.05 | 0.08 | 0.1 |
| 4 | 0.05 | 1×10^-12^ | 0.5 |
| 5 | 0.4 | 2×10^-5^ | 0.8 |
| 6 | 0.001 | 0.01 | 0.2 |
| 7 | 0.2 | 0.001 | 0.4 |
| 8 | 0.1 | 0.07 | 0.07 |


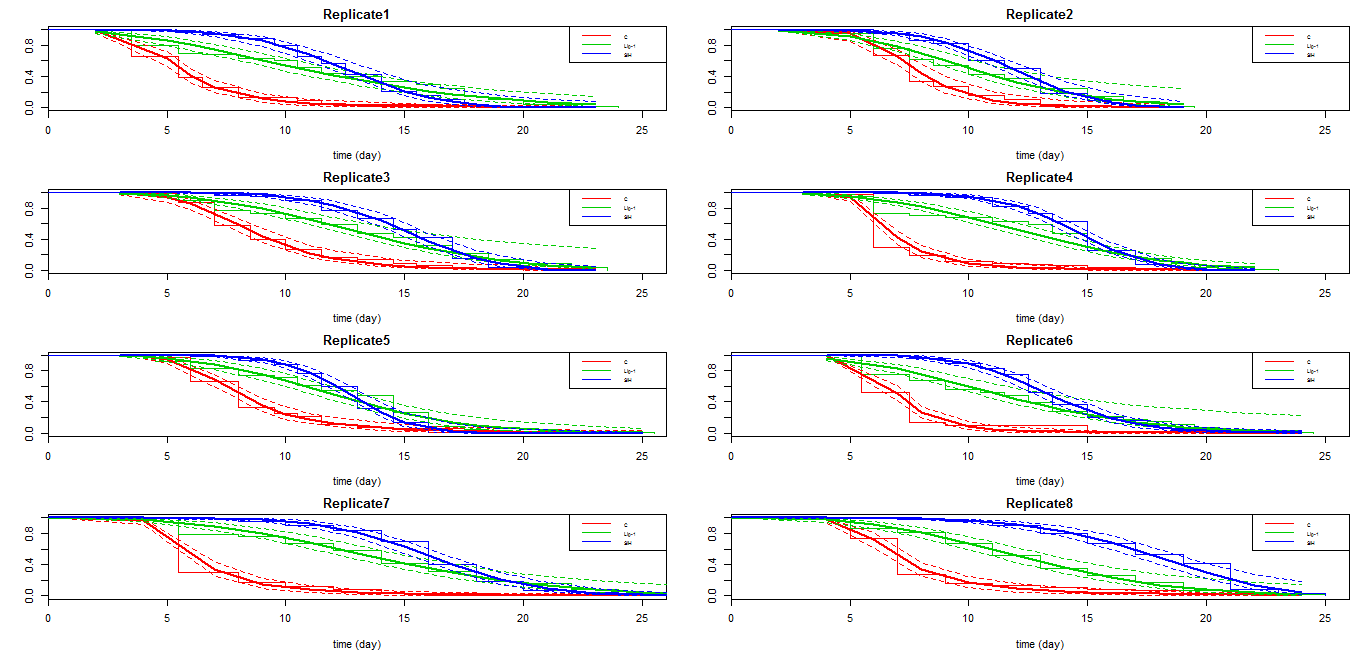


**Figure 3:** The observed and fitted survival curves based on the best non-AFT model for each replicate

**Combining Replicates**

**Models for Combining Similar Replicates**

We tried to identify the most parsimonious model that can best fit the combined data from all replicates. If some of the parameters are quite similar across replicates, we can fit a simpler model than the saturated model where all parameters are allowed to be different across replicates. A range of models are fitted and the best simple model for the combined data is Model 6 (M6) with the same treatment-dependent shapes and heterogeneity parameters across replicates but replicate-specific parameters for scale parameter of the control worms and temporal rescaling parameters. The BIC value for this model is smaller than that for the saturated model (15079 *vs* 15347) and the LR test statistic is 79.2 (df = 90) with *p*-value = 0.78, indicating that based on LR test the combined model (M6) does not provide worse fit to the data. The need for replicate-specific scale parameters and temporal rescaling parameters corroborates the evidence in **Figure 3** which showed these parameters as having considerable variations across replicates.

Goodness-of-fit (GOF) test at replicate-level based on model M6 (**Table 4**) shows that this model provides more or less the same level of fit to the replicate-specific model (**Table 3**), with replicates showing good fit before still showing good fit now. The fitted survival curves (based on M6) are given in **Figure 4**.

**Table 4:** Chi-square Goodness of Fit Statistics (*p*-values) for M6 (the best parsimonious model according to BIC)

| Replicate | Control | Lip-1 | SIH |
| --- | --- | --- | --- |
| 1 | 6 x 10^-1^ | 10^-6^ | 5 x 10^-1^ |
| 2 | 2 x 10^-1^ | 5 x 10^-2^ | 4 x 10^-2^ |
| 3 | 9 x 10^-3^ | 10^-1^ | 2 x 10^-1^ |
| 4 | 10^-2^ | 4 x 10^-11^ | 2 x 10^-1^ |
| 5 | 5 x 10^-1^ | 4 x 10^-5^ | 4 x 10^-1^ |
| 6 | 3 x 10^-7^ | 10^-2^ | 4 x 10^-2^ |
| 7 | 3 x 10^-1^ | 3 x 10^-3^ | 5 x 10^-1^ |
| 8 | 2 x 10^-1^ | 2 x 10^-1^ | 7 x 10^-2^ |


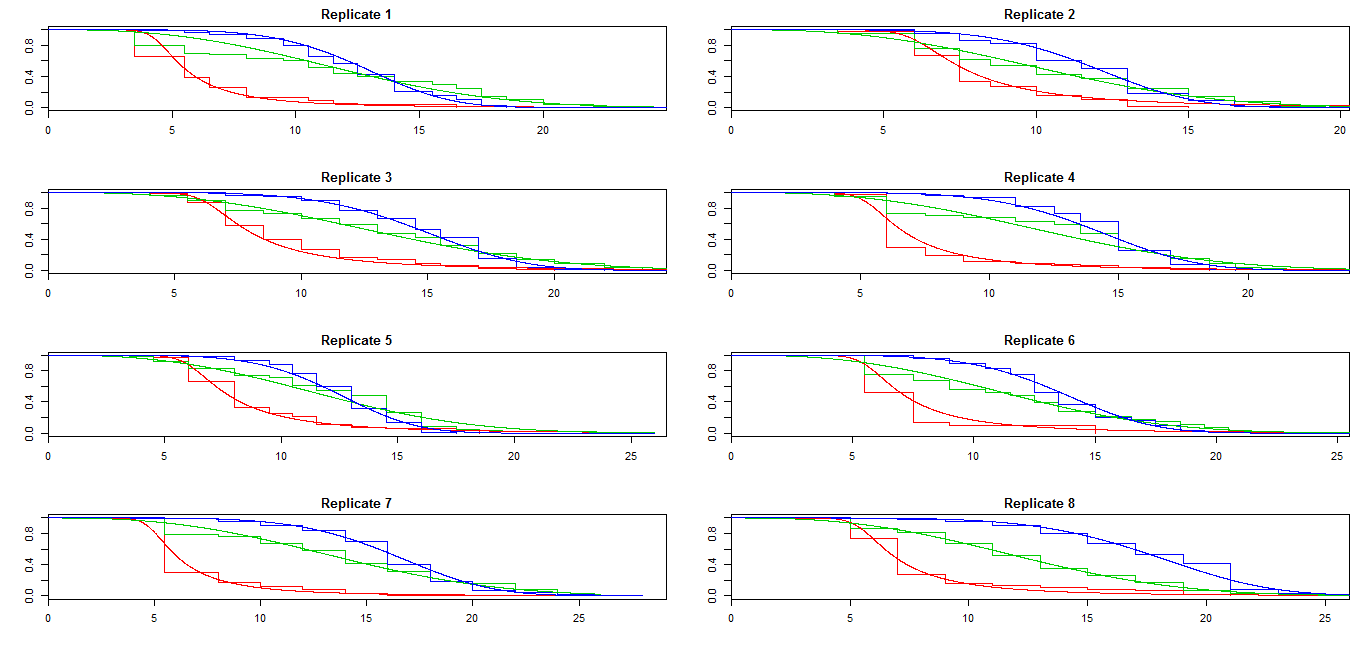


**Figure 4**: The observed and fitted survival curves based on model the most parsimonious model M6

**Meta-Analysis**

In **Table 5**, we present the parameter estimates from the best non-AFT model with Weibull frailty hazards for each replicate and also fixed-effect and random-effect meta-analysis estimates for each parameter of the model. Briefly, the fixed-effect meta-analysis estimates were derived using Inverse Variance Weighting (IVW) in which the estimates from each replicate were weighted by the inverse of the variance estimates. The meta-analysis estimates were then calculated simply as the weighted average of estimates from all replicates. The fixed-effect meta-analysis assumes there is insignificant variation in the estimates of the same parameter across different replicates. The random-effect meta-analysis derived the estimates by also assigning weights to estimates from each replicate, but the weights take into account the variation of estimates across replicates.


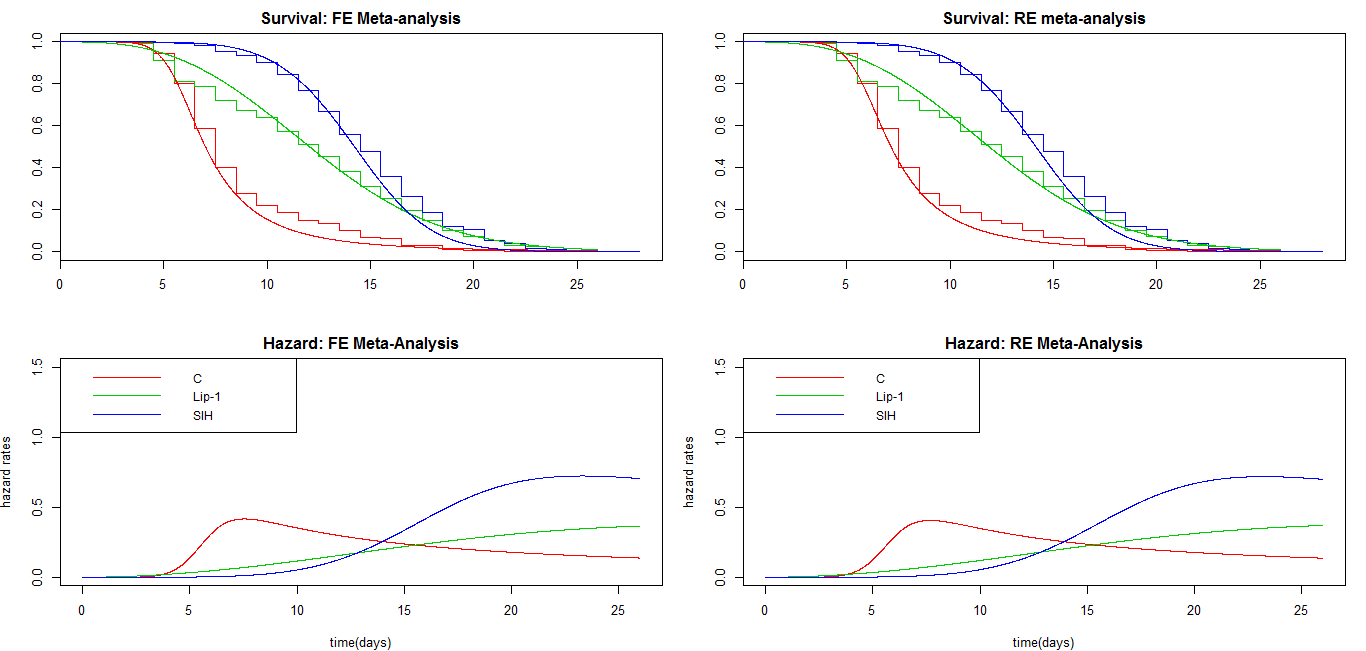


**Figure 5**: Meta-analysis Estimates of Survival and Hazard Functions. FE=fixed error, RE=random error.

**Table 5**: Parameter Estimates and their 95% confidence intervals from non-AFT Weibull-Frailty Models with treatment-dependent shape and heterogeneity parameters

| Replicate | **log(a_C_)** | **log(b_C_)** | **log(σ^2^_C_)** | **θ^1^_Lip-1_** | **θ^1^_SIH_** | Δlog(a_Lip-1_) | Δlog(a_SIH_) | Δlog(σ^2^_Lip-1_) | Δlog(σ^2^_SIH_) |
| --- | --- | --- | --- | --- | --- | --- | --- | --- | --- |
| **1** | 1.73(1.1;2.36) | 1.67(1.55;1.8) | 0.24(-0.24;0.71) | 2.37(2.01;2.8) | 2.5(2.16;2.89) | -1.02(-1.67;-0.36) | -0.17(-0.85;0.51) | -1.67(-2.91;-0.44) | -1.15(-2.35;0.05) |
| **2** | 1.9(1.25;2.55) | 2.04(1.91;2.17) | 0.04(-0.61;0.7) | 1.41(1.13;1.76) | 1.62(1.4;1.88) | -0.74(-1.48;-0.01) | -0.23(-0.92;0.47) | -0.57(-1.84;0.69) | -0.79(-1.91;0.32) |
| **3** | 1.65(1.13;2.17) | 2.14(2;2.29) | 0.03(-0.55;0.61) | 1.7(1.42;2.04) | 1.86(1.6;2.17) | -0.54(-1.09;0.02) | 0.16(-0.39;0.72) | -1.12(-2.75;0.51) | -0.97(-2.01;0.07) |
| **4** | 2.47(2.04;2.9) | 1.84(1.77;1.9) | 0.5(0.22;0.78) | 2.19(1.99;2.4) | 2.4(2.23;2.59) | -1.39(-1.84;-0.93) | -0.5(-0.97;-0.03) | -1.66(-2.48;-0.83) | -1.24(-1.94;-0.53) |
| **5** | 1.91(1.42;2.39) | 2.04(1.92;2.16) | 0.27(-0.17;0.71) | 1.71(1.48;1.98) | 1.72(1.52;1.96) | -0.72(-1.25;-0.2) | 0.08(-0.45;0.62) | -1.18(-2.07;-0.29) | -1.05(-2.06;-0.04) |
| **6** | 2.76(2.06;3.46) | 1.9(1.85;1.94) | 0.56(0.19;0.94) | 1.88(1.62;2.18) | 2.04(1.88;2.21) | -1.73(-2.47;-0.99) | -0.81(-1.57;-0.05) | -1.42(-2.75;-0.09) | -0.75(-1.34;-0.17) |
| **7** | 2.26(1.76;2.76) | 1.74(1.62;1.85) | 0.53(0.18;0.88) | 2.69(2.3;3.15) | 2.94(2.58;3.34) | -1.23(-1.77;-0.69) | -0.42(-0.98;0.14) | -1.44(-2.34;-0.53) | -0.93(-1.51;-0.34) |
| **8** | 2.19(1.7;2.69) | 1.86(1.77;1.96) | 0.51(0.17;0.85) | 2.08(1.78;2.42) | 2.96(2.66;3.3) | -1.08(-1.64;-0.53) | -0.52(-1.05;0.02) | -1.31(-2.35;-0.27) | -1.83(-3.03;-0.63) |
| **Meta-analysis (Fixed Effects)**  **ALL** | 2.12(1.93;2.30) | 1.88(1.85;1.91) | 0.42(0.28;0.56) | 2.04(1.94;2.15) | 2.26(2.17;2.35) | -1.05(-1.26;-0.85) | -0.29(-0.49;-0.08) | -1.35(-1.72;-0.97) | -1.01(-1.3;-0.72) |
| **Meta-analysis (Random Effects)**  **ALL** | 2.11(1.86;2.36) | 1.90(1.79;2.00) | 0.42(0.28;0.56) | 1.97(1.73;2.27) | 2.20(1.88;2.59) | -1.05(-1.31;-0.79) | -0.29(-0.51;-0.06) | -1.35(-1.72;-0.97) | -1.01(-1.3;-0.72) |

^1^θ_Lip-1_ = b_Lip-1_/b_C_ ; θ_SIH_ = b_SIH_/b_C_

Conclusions from **Table 5**:

- **There are between-replicate variations in the scaling factor estimates. But after combining across different replicates, θ_SIH_** estimate is 2.20(95% CI: 1.88;2.59) and **θ_Lip-1_** estimate is 1.97(95% CI: 1.73;2.27), which means that life has significantly de-accelerated under Lip-1 and SIH relative to under controls, by about half.
- **Meta-analysis estimates also shows** that the treatment effect manifests in not only simple scaling of the Weibull scale parameter, but it also affects the shape parameters with the shape parameters for Lip-1 and SIH being significantly smaller (the 95% CI for differences in log shape parameter between Lip-1 and SIH versus controls do not include zero).
- **Meta-analysis estimates also shows** there is significant heterogeneity due to unobserved factors among control worms, 95% CI for log σ^2^_c_ = 0.42(95% CI: 0.28;0.56) while the heterogeneity due to unobserved factors are significantly less in Lip-1 and SIH worms.

**Table 6**: Comparison of Models for Combined Data

| **Model** | **log(a)** | **log(b)** | **log(σ^2^_C_)** | **θ_Lip-1_** | **θ_SIH_** | **Δlog(a_Lip-1_)** | **Δlog(a_SIH_)** | **Δlog(σ^2^_Lip-1_)** | **Δlog(σ^2^_SIH_)** | **BIC value** |
| --- | --- | --- | --- | --- | --- | --- | --- | --- | --- | --- |
| M1 | same | different | same | same | same | 0 | 0 | 0 | 0 | 15835.49 |
| M2 | same | different | same | different | different | 0 | 0 | 0 | 0 | 15887.84 |
| M3 | same | different | same | same | same | 0 | 0 | same | same | 15490.08 |
| M4 | same | different | same | different | different | 0 | 0 | same | same | 15511.38 |
| M5 | same | different | same | same | same | same | same | same | same | 15109.10 |
| **M6** | **same** | **different** | **same** | **different** | **different** | **same** | **same** | **same** | **same** | **15079.13** |
| M7 | different | different | different | different | different | same | same | same | same | 15250.78 |
| Saturated | different | different | different | different | different | different | different | different | different | 15346.79 |

**same = assumed the same across replicates**

**different = allowed to be different across replicates**

1. **= not estimated, set to zero**

**
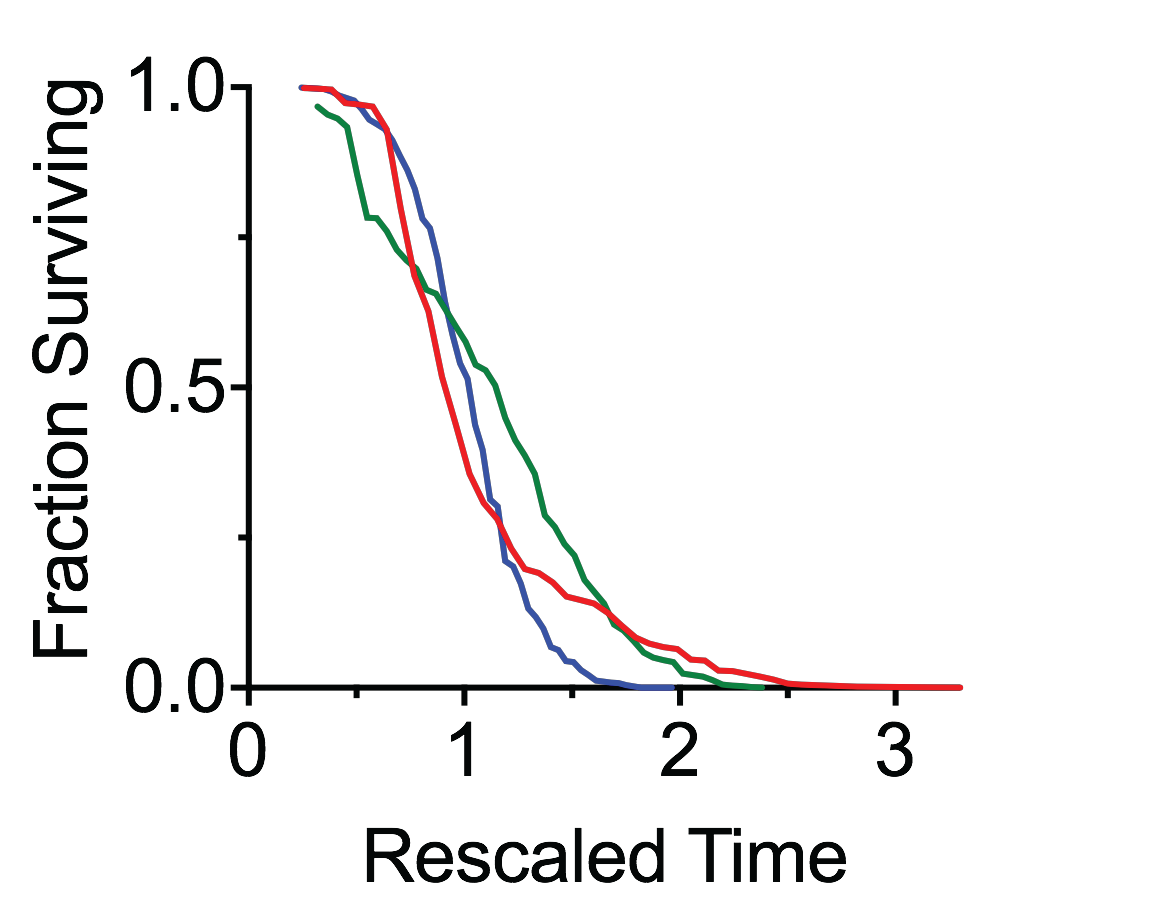
**

**Figure 6**: The best-fit AFT residuals from survival data meta-analysis shown in Figure 5, based the M6 model with fixed errors. Note that the survival curves cross rather than overlap, indicating temporal scaling is insufficient to explain the differences between treatments

**Investigating Temporal Rescaling by Temperature**

To investigate if changing the temperature showed evidence of temporal scaling we compared worms aged at 20 °C and 25 °C during the same time frame. The effect of temperature is evaluated separately for control and SIH treated worms, population sizes for each replicate are shown in **Table 7**.

**Table 7:** Population sizes for replicates used to assess temporal rescaling by temperature

| Replicate | Temperature | **control** | **SIH** |
| --- | --- | --- | --- |
|  |  | death events | death events |
| 1 | 20 °C | 99 | 100 |
| 1 | 25 °C | 76 | 90 |
| 2 | 20 °C | 84 | 80 |
| 2 | 25 °C | 81 | 67 |

**Checking AFT Assumption**

For each of two replicates and treatment factors, the AFT model with Weibull baseline hazard was fitted with temperature as the covariate. The residuals from this model were then subjected to the K-S test. The *p*-values from the K-S test are given in **Table 8**. As can be seen, the *p*-values are generally not very small (only one *p*-value < 0.01), indicating that the evidence of departure from simple temporal rescaling is not strong. **Figure 7** shows the survival curves of the residuals and despite the discreteness, the two distributions (black for 20 °C and red for 25 °C) seem to be similar.

**Table 8:** *p*-values of KS test on Residuals of nonparametric AFT models

| **Replicate** | **Treatment** | ***p*-value** |
| --- | --- | --- |
| 1 | Control | 2.0 x 10^-2^ |
| 1 | SIH | 3.5 x 10^-2^ |
| 2 | Control | 2.7 x 10^-3^ |
| 2 | SIH | 1.3 x 10^-1^ |

**Figure 7**: The best-fit AFT residuals from the model with Weibull baseline hazard for survival of control and SIH treated worms at 20 °C (black) and 25 °C (red)

**Meta-Analysis**

We also performed meta-analysis for each worm population and the results are given below. The log σ^2^ provides an indication of the level of heterogeneity, and it is interesting to note that the control worms exhibit greater heterogeneity than the SIH worms, consistent with that observed at 25 °C.

For both populations, being exposed to the higher temperature of 25 °C accelerates life as expected, by approximately 30% for control worms (θ_temp_ = **0.72 (95% CI: 0.68;0.77)** and 20% for SIH worms (θ_temp_ = **0.81 (95% CI: 0.78;0.84)**.

**Table 9**: Meta-analysis results for control and SIH treated worms

| ***Control worms*** | | | | |
| --- | --- | --- | --- | --- |
| **Replicate** | **Log Shape** | **Log Scale** | **Log σ^2^** | **θ_temp_ (25 *vs* 20°C)** |
| 1 | 2.00(1.65;2.34) | 2.48(2.37;2.60) | 0.35(0.03;0.66) | 0.66(0.60;0.73) |
| 2 | 3.09(2.37;3.82) | 2.16(2.04;2.28) | 1.11(0.70;1.52) | 0.79(0.72;0.86) |
| Meta-Analysis | 2.20(1.89;2.51) | 2.32(2.24;2.41) | 0.63(0.38;0.88) | **0.72(0.68;0.77)** |
|  |  |  |  |  |
| **SIH *treated*** | | | | |
| **Replicate** | **Log Shape** | **Log Scale** | **Log σ^2^** | **θ_temp_ (25 *vs* 20°C)** |
| 1 | 1.70(1.58;1.81) | 2.93(2.89;2.97) | -3.93(-18.49;10.63) | 0.76(0.72;0.81) |
| 2 | 1.87(1.64;2.11) | 3.06(3.01;3.12) | -0.84(-1.90;0.21) | 0.87(0.82;0.93) |
| Meta-Analysis | 1.73(1.63;1.83) | 2.97(2.94;3.00) | -0.86(-1.91;0.19) | **0.81(0.78;0.84)** |

**References**

Buckley, J., and James, I.A.N. (1979). Linear regression with censored data. Biometrika *66*, 429-436.

Fleming, T.R., O'Fallon, J.R., O'Brien, P.C., and Harrington, D.P. (1980). Modified Kolmogorov-Smirnov Test Procedures with Application to Arbitrarily Right-Censored Data. Biometrics *36*, 607-625.

Lindsey, J.C., and Ryan, L.M. (1998). Methods for interval-censored data. Statistics in Medicine *17*, 219-238.

Stroustrup, N., Anthony, W.E., Nash, Z.M., Gowda, V., Gomez, A., Lopez-Moyado, I.F., Apfeld, J., and Fontana, W. (2016). The temporal scaling of *Caenorhabditis elegans* ageing. Nature *530*, 103-107.
