# Supplementary material for: Changes in ferrous iron and glutathione promote ferroptosis and frailty in aging Caenorhabditis elegans
Source: eLife. 2020 Jul 21;9:e56580. doi: 10.7554/eLife.56580 (PMC7373428; doi:10.7554/eLife.56580)
Supplement: Supplementary file 6. [file elife-56580-supp6.docx]

**Body length analysis**

Summary statistics and tests for normality of body length are included in **Table 1**. Not all length or derived data sets were normally distributed, as indicated below.

**Table 1**: Summary of body length comparisons between treatments and ages

|  | Start | Day 1 Control | Day 1 Lip-1 | Day 1 SIH | Day 4 Control | Day 4 Lip-1 | Day 4 SIH | Day 8 Control | Day 8 Lip-1 | Day 8 SIH |
| --- | --- | --- | --- | --- | --- | --- | --- | --- | --- | --- |
| Number of values | 125 | 82 | 98 | 90 | 71 | 49 | 68 | 17 | 10 | 21 |
|  |  |  |  |  |  |  |  |  |  |  |
| Minimum | 876.2 | 1159 | 1114 | 1024 | 934.2 | 1023 | 1377 | 1229 | 1171 | 1532 |
| 25% Percentile | 979.9 | 1341 | 1319 | 1371 | 1317 | 1277 | 1563 | 1346 | 1290 | 1651 |
| Median | 1026 | 1387 | 1364 | 1412 | 1432 | 1345 | 1614 | 1403 | 1463 | 1704 |
| 75% Percentile | 1084 | 1432 | 1403 | 1443 | 1519 | 1445 | 1648 | 1561 | 1524 | 1742 |
| Maximum | 1271 | 1527 | 1488 | 1521 | 1660 | 1589 | 1773 | 1619 | 1554 | 1815 |
|  |  |  |  |  |  |  |  |  |  |  |
| Mean | 1036 | 1387 | 1358 | 1400 | 1414 | 1344 | 1605 | 1440 | 1414 | 1696 |
| Std. Deviation | 80.61 | 67.69 | 66.66 | 80.33 | 146.2 | 128.5 | 77.57 | 122.7 | 133.9 | 63.91 |
| Std. Error of Mean | 7.21 | 7.475 | 6.734 | 8.467 | 17.35 | 18.36 | 9.406 | 29.76 | 42.33 | 13.95 |
|  |  |  |  |  |  |  |  |  |  |  |
| Lower 95% CI of mean | 1022 | 1372 | 1345 | 1383 | 1379 | 1308 | 1586 | 1376 | 1318 | 1667 |
| Upper 95% CI of mean | 1051 | 1402 | 1372 | 1417 | 1448 | 1381 | 1624 | 1503 | 1509 | 1725 |
| D'Agostino & Pearson normality test | | | | | | | | | | |
| K2 | 6.981 | 5.546 | 18.41 | 63.3 | 8.069 | 5.393 | 7.285 | 3.174 | 1.47 | 1.822 |
| *p* value | 0.0305 | 0.0625 | 0.0001 | <0.0001 | 0.0177 | 0.0675 | 0.0262 | 0.2045 | 0.4794 | 0.4021 |
| **Passed normality test (α=0.05)?** | **No** | **Yes** | **No** | **No** | **No** | **Yes** | **No** | **Yes** | **Yes** | **Yes** |
| *p* value summary | * | ns | *** | **** | * | ns | * | ns | ns | ns |

To compare between age and treatment groups a Kruskal-Wallace ANOVA was performed, followed by Dunn’s multiple comparisons Post-hoc tests. There was a significant difference between body length (H(10)= 432.6, *p* < 0.0001) amongst the groups measured. The results of the pairwise comparisons, corrected for multiple comparisons, are shown in **Table 2.**

**Table 2:** Summary of body length comparisons between ages and treatments.

| Dunn's multiple comparisons test | Mean rank diff. | Significant? | Adjusted *p* Value |
| --- | --- | --- | --- |
| Start vs. Day 1 Control | -261.3 | Yes | <0.0001 |
| Start vs. Day 1 Lip-1 | -219.1 | Yes | <0.0001 |
| Start vs. Day 1 SIH | -305.1 | Yes | <0.0001 |
| Day 1 Control vs. Day 1 Lip-1 | 42.24 | No | >0.9999 |
| Day 1 Control vs. Day 1 SIH | -43.77 | No | >0.9999 |
| Day 1 Control vs. Day 4 Control | -44.11 | No | >0.9999 |
| Day 4 Control vs. Day 4 Lip-1 | 84.76 | No | 0.1743 |
| Day 4 Control vs. Day 4 SIH | -184 | Yes | <0.0001 |
| Day 4 Control vs. Day 8 Control | -7.775 | No | >0.9999 |
| Day 8 Control vs. Day 8 Lip-1 | 9.976 | No | >0.9999 |
| Day 8 Control vs. Day 8 SIH | -223.5 | Yes | 0.0023 |
| Day 1 Lip-1 vs. Day 4 Lip-1 | -1.591 | No | >0.9999 |
| Day 4 Lip-1 vs. Day 8 Lip-1 | -82.56 | No | >0.9999 |
| Day 1 SIH vs. Day 4 SIH | -184.4 | Yes | <0.0001 |
| Day 4 SIH vs. Day 8 SIH | -47.25 | No | >0.9999 |

**Volume analysis**

Summary statistics and tests for normality of total worm volume are included in **Table 3**. Not all length or derived data sets were normally distributed, as indicated below.

**Table 3**: Summary of volume comparisons between treatments and ages

|  | Start | Day 1 Control | Day 1 Lip-1 | Day 1 SIH | Day 4 Control | Day 4 Lip-1 | Day 4 SIH | Day 8 Control | Day 8 Lip-1 | Day 8 SIH |
| --- | --- | --- | --- | --- | --- | --- | --- | --- | --- | --- |
| Number of values | 125 | 82 | 98 | 90 | 71 | 49 | 68 | 17 | 10 | 21 |
|  |  |  |  |  |  |  |  |  |  |  |
| Minimum | 747.8 | 3115 | 2740 | 1731 | 3293 | 2258 | 4052 | 4939 | 4203 | 5461 |
| 25% Percentile | 1589 | 4051 | 3883 | 4057 | 5049 | 4251 | 6677 | 5655 | 4882 | 7280 |
| Median | 1867 | 4247 | 4293 | 4393 | 6103 | 5032 | 7183 | 6673 | 6147 | 7726 |
| 75% Percentile | 2083 | 4576 | 4598 | 4740 | 6507 | 5648 | 7738 | 7465 | 6752 | 8407 |
| Maximum | 3818 | 5249 | 5420 | 5800 | 8267 | 6712 | 8608 | 8696 | 7876 | 9210 |
|  |  |  |  |  |  |  |  |  |  |  |
| Mean | 1901 | 4289 | 4209 | 4369 | 5810 | 4970 | 7111 | 6511 | 5958 | 7731 |
| Std. Deviation | 495.1 | 417.5 | 540.8 | 643.1 | 1111 | 965.4 | 861.8 | 1043 | 1155 | 825.4 |
| Std. Error of Mean | 44.28 | 46.1 | 54.63 | 67.79 | 131.8 | 137.9 | 104.5 | 252.9 | 365.2 | 180.1 |
|  |  |  |  |  |  |  |  |  |  |  |
| Lower 95% CI of mean | 1813 | 4197 | 4101 | 4234 | 5547 | 4693 | 6903 | 5975 | 5132 | 7355 |
| Upper 95% CI of mean | 1988 | 4380 | 4318 | 4504 | 6073 | 5248 | 7320 | 7047 | 6784 | 8106 |
|  |  |  |  |  |  |  |  |  |  |  |
| D'Agostino & Pearson normality test | | | | | | | | | | |
| K2 | 38.42 | 0.4213 | 2.657 | 37.57 | 2.331 | 1.45 | 12.99 | 0.3558 | 0.1231 | 4.164 |
| *p* value | <0.0001 | 0.8101 | 0.2649 | <0.0001 | 0.3118 | 0.4843 | 0.0015 | 0.8370 | 0.9403 | 0.1247 |
| Passed normality test (α=0.05)? | No | Yes | Yes | No | Yes | Yes | No | Yes | Yes | Yes |
| *p* value summary | **** | ns | ns | **** | ns | ns | ** | ns | ns | ns |

There was a significant difference between body volume (H(10)= 489, *p* < 0.0001) amongst the groups measured. Comparisons between age and treatment groups a Kruskal-Wallace ANOVA was performed, followed by Dunn’s multiple comparisons Post-hoc tests. The results of the pairwise comparisons, corrected for multiple comparisons, are shown in **Table 4**.

**Table 4:** Summary of volume comparisons between ages and treatments.

| Dunn's multiple comparisons test | Mean rank diff. | Significant? | Adjusted *p* Value |
| --- | --- | --- | --- |
|  |  |  |  |
| Start vs. Day 1 Control | -214.9 | Yes | <0.0001 |
| Start vs. Day 1 Lip-1 | -205.6 | Yes | <0.0001 |
| Start vs. Day 1 SIH | -236.4 | Yes | <0.0001 |
| Day 1 Control vs. Day 1 Lip-1 | 9.301 | No | >0.9999 |
| Day 1 Control vs. Day 1 SIH | -21.45 | No | >0.9999 |
| Day 1 Control vs. Day 4 Control | -175 | Yes | <0.0001 |
| Day 4 Control vs. Day 4 Lip-1 | 80.42 | No | 0.2630 |
| Day 4 Control vs. Day 4 SIH | -102.6 | Yes | 0.0136 |
| Day 4 Control vs. Day 8 Control | -66.73 | No | >0.9999 |
| Day 8 Control vs. Day 8 Lip-1 | 54.78 | No | >0.9999 |
| Day 8 Control vs. Day 8 SIH | -70.02 | No | >0.9999 |
| Day 1 Lip-1 vs. Day 4 Lip-1 | -103.9 | Yes | 0.0169 |
| Day 4 Lip-1 vs. Day 8 Lip-1 | -92.37 | No | >0.9999 |
| Day 1 SIH vs. Day 4 SIH | -256.2 | Yes | <0.0001 |
| Day 4 SIH vs. Day 8 SIH | -34.11 | No | >0.9999 |

Summary statistics and tests for normality of maximum velocity (mm s^-1^) are included in **Table 5**. Not all data sets were normally distributed, as indicated below.

**Table 5**: Summary of maximum velocity results across treatments and ages

|  | Day 1 Control | Day 4 Control | Day 8 Control | Day 4 SIH | Day 8 SIH | Day 4 Lip-1 | Day 8 Lip-1 |
| --- | --- | --- | --- | --- | --- | --- | --- |
| Number of values | 103 | 82 | 73 | 99 | 83 | 105 | 104 |
|  |  |  |  |  |  |  |  |
| Minimum | 0.1832 | 0.04309 | 0.03738 | 0.105 | 0.1028 | 0.06351 | 0.05622 |
| 25% Percentile | 0.3731 | 0.1294 | 0.06372 | 0.3442 | 0.174 | 0.2711 | 0.1478 |
| Median | 0.4509 | 0.2304 | 0.09115 | 0.3733 | 0.2513 | 0.3762 | 0.1922 |
| 75% Percentile | 0.5491 | 0.3364 | 0.1254 | 0.4755 | 0.3506 | 0.4861 | 0.2452 |
| Maximum | 0.8264 | 0.6134 | 0.3802 | 0.7127 | 0.6458 | 0.7204 | 0.5563 |
|  |  |  |  |  |  |  |  |
| Mean | 0.4692 | 0.2536 | 0.109 | 0.392 | 0.2847 | 0.37 | 0.2084 |
| Std. Deviation | 0.1457 | 0.1496 | 0.06568 | 0.1087 | 0.1356 | 0.1588 | 0.09062 |
| Std. Error of Mean | 0.01435 | 0.01653 | 0.007687 | 0.01093 | 0.01489 | 0.01549 | 0.008886 |
|  |  |  |  |  |  |  |  |
| Lower 95% CI of mean | 0.4408 | 0.2207 | 0.09367 | 0.3704 | 0.2551 | 0.3393 | 0.1908 |
| Upper 95% CI of mean | 0.4977 | 0.2865 | 0.1243 | 0.4137 | 0.3143 | 0.4007 | 0.226 |
| D'Agostino & Pearson normality test | | | | | | | |
| K2 | 4.785 | 8.157 | 44.36 | 0.9424 | 10.76 | 3.145 | 23.3 |
| *p* value | 0.0914 | 0.0169 | <0.0001 | 0.6242 | 0.0046 | 0.2075 | <0.0001 |
| Passed normality test (α=0.05)? | Yes | No | No | Yes | No | Yes | No |
| *p* value summary | ns | * | **** | ns | ** | ns | **** |

To compare between age and treatment groups a Kruskal-Wallace ANOVA was performed, followed by Dunn’s multiple comparisons post-hoc tests. There was a significant difference between maximum velocity (H(7)=298.5, *p* < 0.0001) amongst the groups measured. The results of the pairwise comparisons, corrected for multiple comparisons, are shown in **Table 6.**

**Table 6:** Summary of maximum velocity comparisons between ages and treatments.

| Dunn's multiple comparisons test | Mean rank diff. | Significant? | Adjusted *p* Value |
| --- | --- | --- | --- |
|  |  |  |  |
| Day 1 Control vs. Day 4 Control | 230.5 | Yes | <0.0001 |
| Day 1 Control vs. Day 8 Control | 411.2 | Yes | <0.0001 |
| Day 4 Control vs. Day 8 Control | 180.7 | Yes | <0.0001 |
| Day 4 Control vs. Day 4 SIH | -168.4 | Yes | <0.0001 |
| Day 4 Control vs. Day 4 Lip-1 | -133.6 | Yes | <0.0001 |
| Day 8 Control vs. Day 8 SIH | -220.9 | Yes | <0.0001 |
| Day 8 Control vs. Day 8 Lip-1 | -132.7 | Yes | <0.0001 |
| Day 4 SIH vs. Day 4 Lip-1 | 34.84 | No | >0.9999 |
| Day 8 SIH vs. Day 8 Lip-1 | 88.29 | Yes | 0.0110 |

**Mean velocity**

Summary statistics and tests for normality of mean velocity (mm s^-1^) are included in **Table 7**. Not all data sets were normally distributed, as indicated below.

**Table 7**: Summary of mean velocity results across treatments and ages

|  | Day 1 Control | Day 4 Control | Day 8 Control | Day 4 SIH | Day 8 SIH | Day 4 Lip-1 | Day 8 Lip-1 |
| --- | --- | --- | --- | --- | --- | --- | --- |
| Number of values | 103 | 82 | 73 | 99 | 83 | 105 | 104 |
|  |  |  |  |  |  |  |  |
| Minimum | 0.04246 | 0.01112 | 0.007567 | 0.02713 | 0.02239 | 0.01219 | 0.008319 |
| 25% Percentile | 0.1382 | 0.02355 | 0.01407 | 0.1307 | 0.04028 | 0.05892 | 0.03386 |
| Median | 0.1856 | 0.04389 | 0.01907 | 0.1545 | 0.05813 | 0.1349 | 0.04763 |
| 75% Percentile | 0.2189 | 0.1139 | 0.02507 | 0.1824 | 0.1029 | 0.1839 | 0.06818 |
| Maximum | 0.2873 | 0.2578 | 0.1104 | 0.2629 | 0.1739 | 0.2606 | 0.1644 |
|  |  |  |  |  |  |  |  |
| Mean | 0.1756 | 0.07449 | 0.02376 | 0.1528 | 0.07262 | 0.1244 | 0.05407 |
| Std. Deviation | 0.05753 | 0.06507 | 0.01791 | 0.0464 | 0.04028 | 0.07094 | 0.02726 |
| Std. Error of Mean | 0.005669 | 0.007186 | 0.002096 | 0.004663 | 0.004421 | 0.006923 | 0.002673 |
|  |  |  |  |  |  |  |  |
| Lower 95% CI of mean | 0.1644 | 0.06019 | 0.01958 | 0.1435 | 0.06382 | 0.1106 | 0.04877 |
| Upper 95% CI of mean | 0.1868 | 0.08878 | 0.02793 | 0.162 | 0.08141 | 0.1381 | 0.05937 |
| D'Agostino & Pearson normality test | | | | | | | |
| K2 | 3.86 | 13.86 | 66.08 | 6.268 | 8.786 | 29.36 | 21.66 |
| *p* value | 0.1451 | 0.0010 | <0.0001 | 0.0435 | 0.0124 | <0.0001 | <0.0001 |
| Passed normality test (α=0.05)? | Yes | No | No | No | No | No | No |
| *p* value summary | ns | *** | **** | * | * | **** | **** |

To compare between age and treatment groups a Kruskal-Wallace ANOVA was performed, followed by Dunn’s multiple comparisons Post-hoc tests. There was a significant difference between mean velocity (H(7)= 339.2, *p* < 0.0001) amongst the groups measured. The results of the pairwise comparisons, corrected for multiple comparisons, are shown in **Table 8.**

**Table 8:** Summary of mean velocity comparisons between ages and treatments.

| Dunn's multiple comparisons test | Mean rank diff. | Significant? | Adjusted *p* Value |
| --- | --- | --- | --- |
|  |  |  |  |
| Day 1 Control vs. Day 4 Control | 259.2 | Yes | <0.0001 |
| Day 1 Control vs. Day 8 Control | 427.8 | Yes | <0.0001 |
| Day 4 Control vs. Day 8 Control | 168.6 | Yes | <0.0001 |
| Day 4 Control vs. Day 4 SIH | -215.1 | Yes | <0.0001 |
| Day 4 Control vs. Day 4 Lip-1 | -135.7 | Yes | <0.0001 |
| Day 8 Control vs. Day 8 SIH | -192.7 | Yes | <0.0001 |
| Day 8 Control vs. Day 8 Lip-1 | -141.2 | Yes | <0.0001 |
| Day 4 SIH vs. Day 4 Lip-1 | 79.45 | Yes | 0.0224 |
| Day 8 SIH vs. Day 8 Lip-1 | 51.53 | No | 0.5569 |

**Total distance travelled**

Summary statistics and tests for normality of total distance travelled (mm) are included in **Table 9**. Not all data were normally distributed, as indicated below.

**Table 9**: Summary of distance travelled results across treatments and ages

|  | Day 1 Control | Day 4 Control | Day 8 Control | Day 4  SIH | Day 8  SIH | Day 4  Lip-1 | Day 8  Lip-1 |
| --- | --- | --- | --- | --- | --- | --- | --- |
| Number of values | 103 | 82 | 73 | 99 | 83 | 105 | 104 |
|  |  |  |  |  |  |  |  |
| Minimum | 1.28 | 0.1883 | 0.2346 | 0.8402 | 0.6912 | 0.07633 | 0.2338 |
| 25% Percentile | 4.197 | 0.7269 | 0.4349 | 3.977 | 1.224 | 1.797 | 1.001 |
| Median | 5.649 | 1.357 | 0.5888 | 4.766 | 1.784 | 4.126 | 1.451 |
| 75% Percentile | 6.388 | 3.521 | 0.7714 | 5.513 | 3.193 | 5.397 | 2.092 |
| Maximum | 8.534 | 7.169 | 3.324 | 6.951 | 5.235 | 7.817 | 4.926 |
|  |  |  |  |  |  |  |  |
| Mean | 5.24 | 2.216 | 0.7247 | 4.619 | 2.192 | 3.685 | 1.627 |
| Std. Deviation | 1.68 | 1.865 | 0.5329 | 1.349 | 1.192 | 2.064 | 0.849 |
| Std. Error of Mean | 0.1656 | 0.2059 | 0.06237 | 0.1356 | 0.1308 | 0.2014 | 0.08325 |
|  |  |  |  |  |  |  |  |
| Lower 95% CI of mean | 4.911 | 1.806 | 0.6003 | 4.35 | 1.932 | 3.285 | 1.462 |
| Upper 95% CI of mean | 5.568 | 2.626 | 0.849 | 4.888 | 2.452 | 4.084 | 1.792 |
| D'Agostino & Pearson normality test | | | | | | | |
| K2 | 4.494 | 10.94 | 63.28 | 9.719 | 8.451 | 27.91 | 16.99 |
| *p* value | 0.1057 | 0.0042 | <0.0001 | 0.0078 | 0.0146 | <0.0001 | 0.0002 |
| Passed normality test (α=0.05)? | Yes | No | No | No | No | No | No |
| *p* value summary | ns | ** | **** | ** | * | **** | *** |

To compare between age and treatment groups a Kruskal-Wallace ANOVA was performed, followed by Dunn’s multiple comparisons Post-hoc tests. There was a significant difference between total distance travelled (H(7)= 340.6, *p*< 0.0001) amongst the groups measured. The results of the pairwise comparisons, corrected for multiple comparisons, are shown in **Table 10.**

**Table 10:** Summary of total distance travelled comparisons between ages and treatments.

| Dunn's multiple comparisons test | Mean rank diff. | Significant? | Adjusted *p* value |
| --- | --- | --- | --- |
| Day 1 Control vs. Day 4 Control | 260.6 | Yes | <0.0001 |
| Day 1 Control vs. Day 8 Control | 425.7 | Yes | <0.0001 |
| Day 4 Control vs. Day 8 Control | 165.1 | Yes | <0.0001 |
| Day 4 Control vs. Day 4 SIH | -218.3 | Yes | <0.0001 |
| Day 4 Control vs. Day 4 Lip-1 | -133.1 | Yes | <0.0001 |
| Day 8 Control vs. Day 8 SIH | -189.4 | Yes | <0.0001 |
| Day 8 Control vs. Day 8 Lip-1 | -134.8 | Yes | <0.0001 |
| Day 4 SIH vs. Day 4 Lip-1 | 85.29 | Yes | 0.0105 |
| Day 8 SIH vs. Day 8 Lip-1 | 54.61 | No | 0.4305 |
