# Supplementary material for: Changes in ferrous iron and glutathione promote ferroptosis and frailty in aging Caenorhabditis elegans
Source: eLife. 2020 Jul 21;9:e56580. doi: 10.7554/eLife.56580 (PMC7373428; doi:10.7554/eLife.56580)
Supplement: Supplementary file 7. — Shown are the masks used to identify and analyse the iron elemental maps of TJ1060 populations at different adult ages ± Lip-1 or SIH at 25°C. (A) Masks for 1 day old adults (starting population) (B) Masks for 4 day old Control adults (C) Masks for 4 day old SIH-treated adults (D) Masks for 4 day old Lip-1 treated adults E: Masks for 8 day old Control adults (F) Masks for 8 day old 250 µM SIH-treated adults (G) Masks for 8 day old 200 µM Lip-1-treated adults. [file elife-56580-supp7.pdf]

A

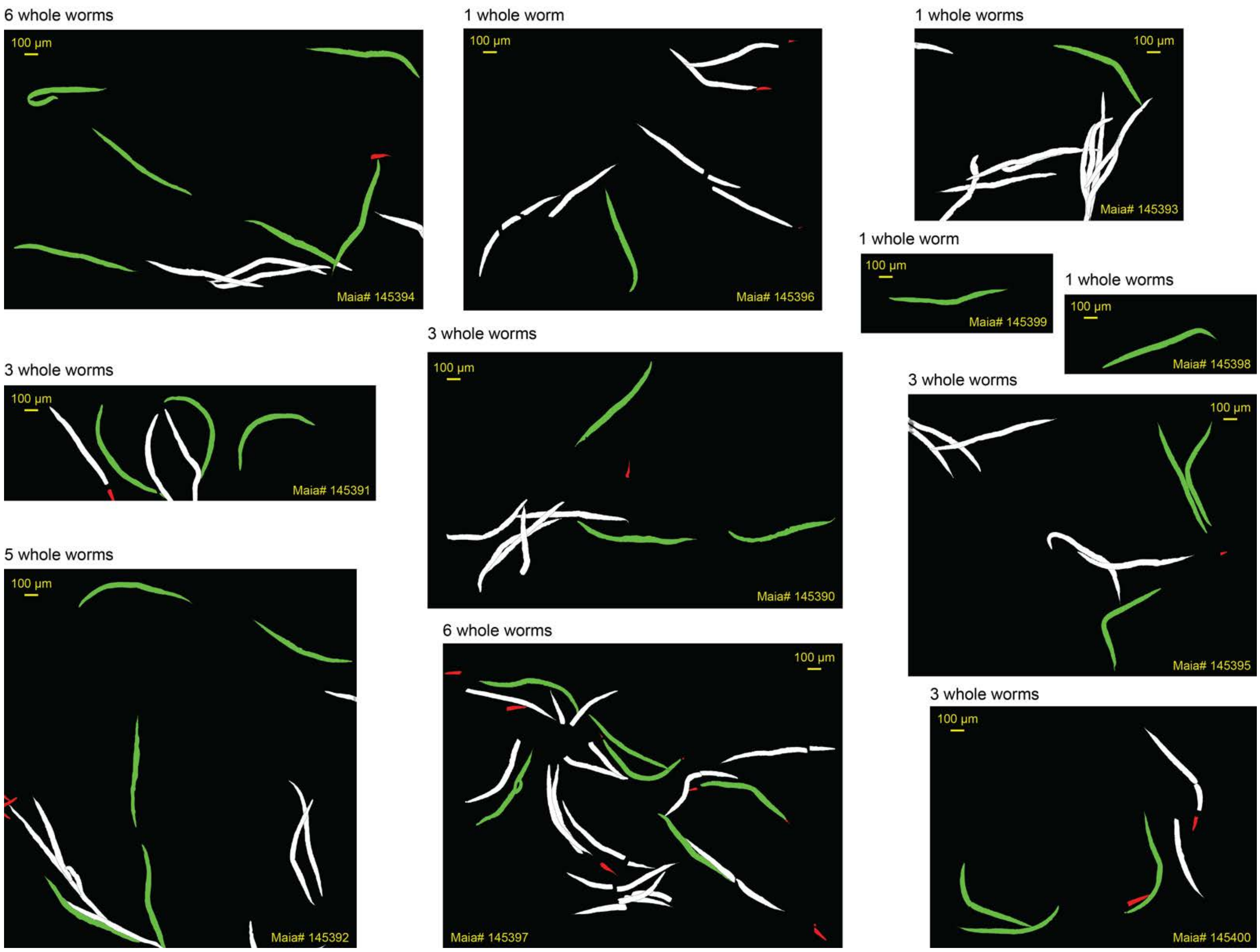
6 whole worms
100 µm
Maia# 145394
1 whole worm
100 µm
Maia# 145396
1 whole worms
100 µm
Maia# 145393
1 whole worm
100 µm
Maia# 145399
1 whole worms
100 µm
Maia# 145398
3 whole worms
100 µm
Maia# 145391
3 whole worms
100 µm
Maia# 145390
3 whole worms
100 µm
Maia# 145395
5 whole worms
100 µm
Maia# 145392
6 whole worms
100 µm
Maia# 145397
3 whole worms
100 µm
Maia# 145400

B

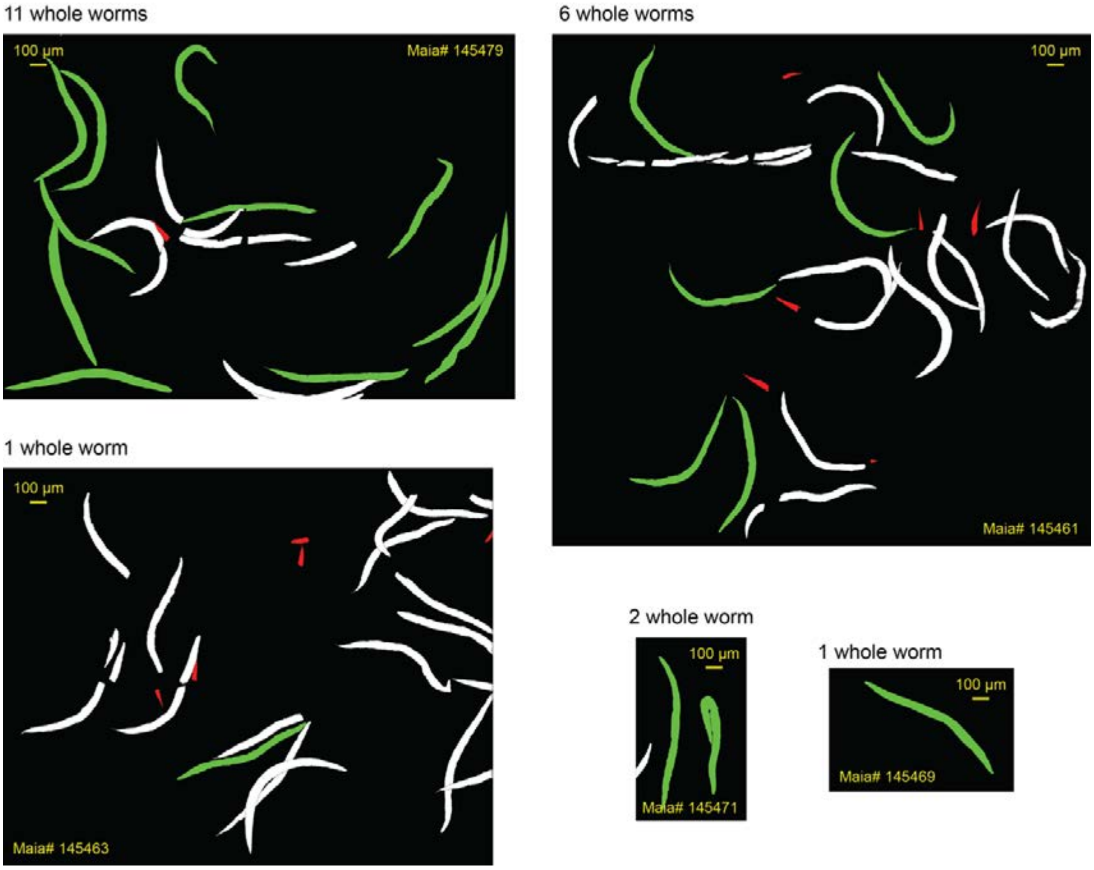
11 whole worms
100 µm
Maia# 145479
6 whole worms
100 µm
Maia# 145461
1 whole worm
100 µm
Maia# 145463
2 whole worm
100 µm
Maia# 145471
1 whole worm
100 µm
Maia# 145469

C

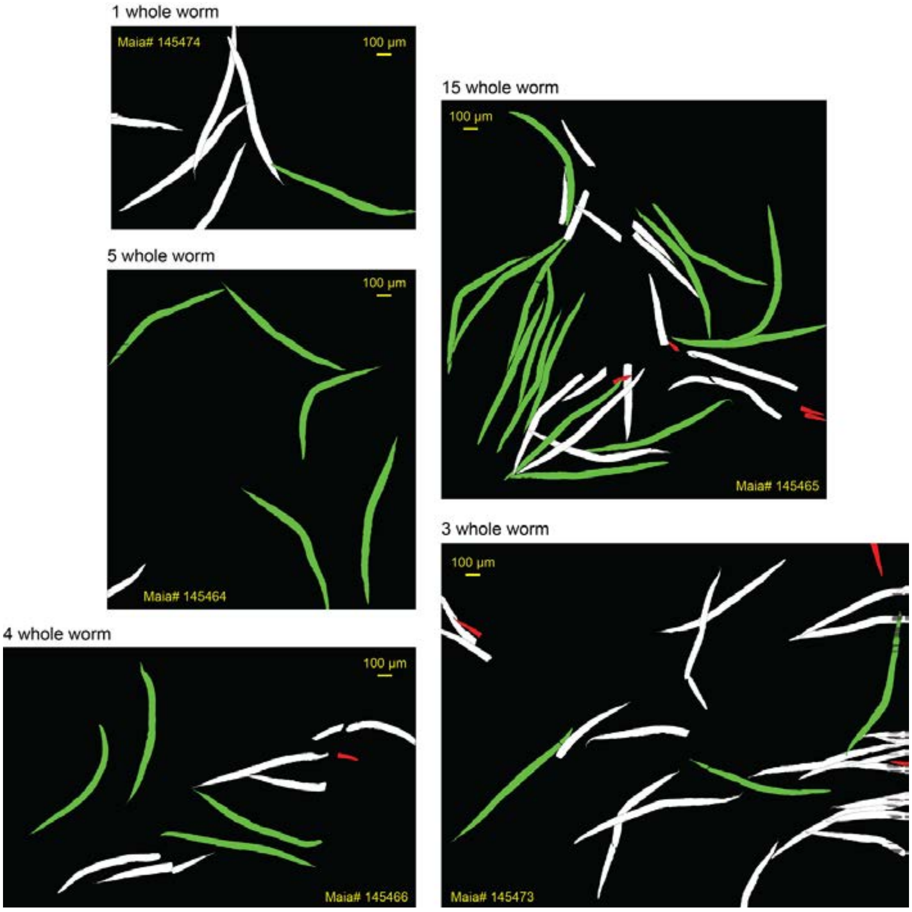
1 whole worm
Maia# 145474
100 µm
15 whole worm
100 µm
Maia# 145465
5 whole worm
100 µm
Maia# 145464
3 whole worm
100 µm
Maia# 145473
4 whole worm
100 µm
Maia# 145466

D

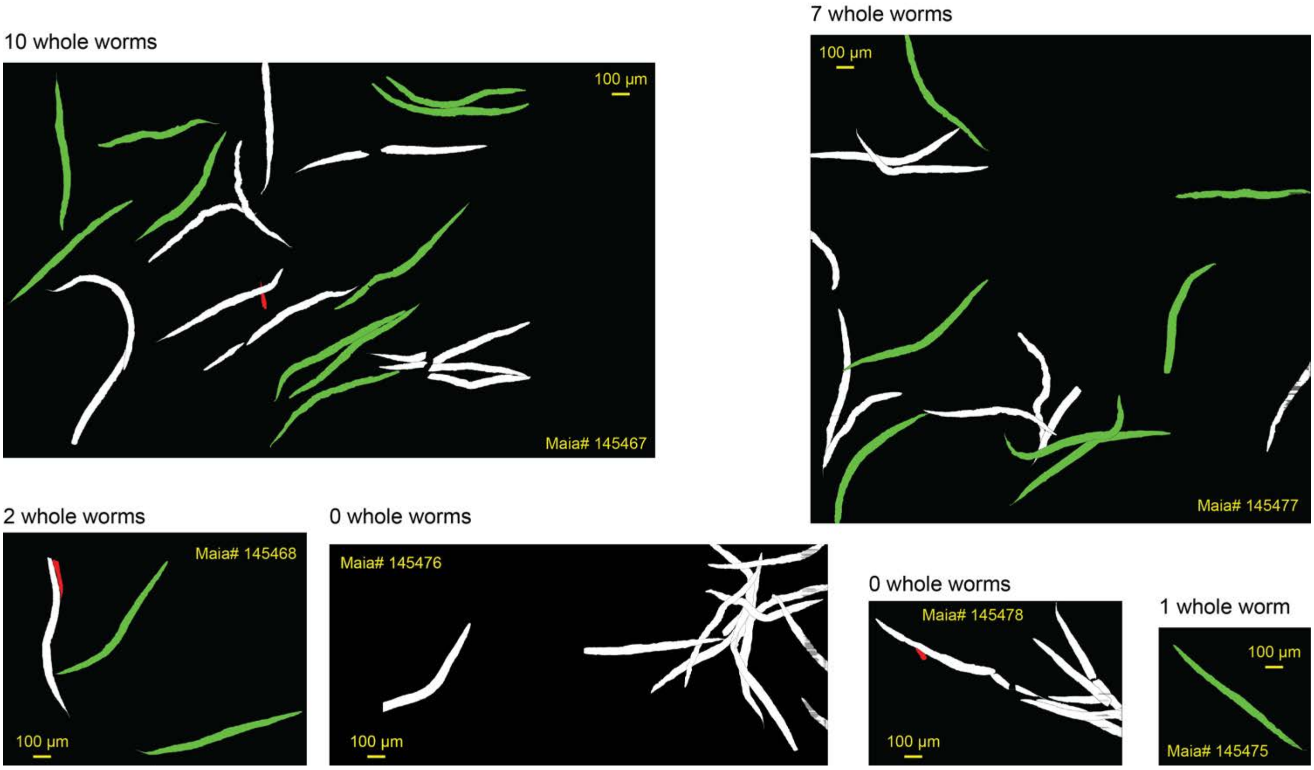

10 whole worms
100 µm
Maia# 145467
7 whole worms
100 µm
Maia# 145477
2 whole worms
Maia# 145468
100 µm
0 whole worms
Maia# 145476
100 µm
0 whole worms
Maia# 145478
100 µm
1 whole worm
100 µm
Maia# 145475

E

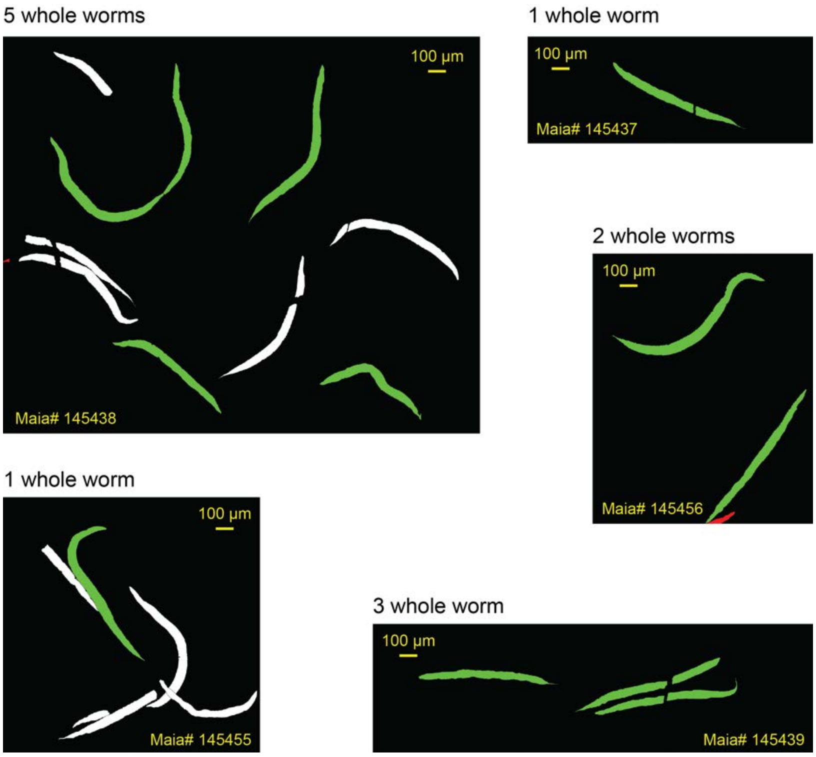

5 whole worms
100 µm
Maia# 145438
1 whole worm
100 µm
Maia# 145437
2 whole worms
100 µm
Maia# 145456
1 whole worm
100 µm
Maia# 145455
3 whole worm
100 µm
Maia# 145439

F

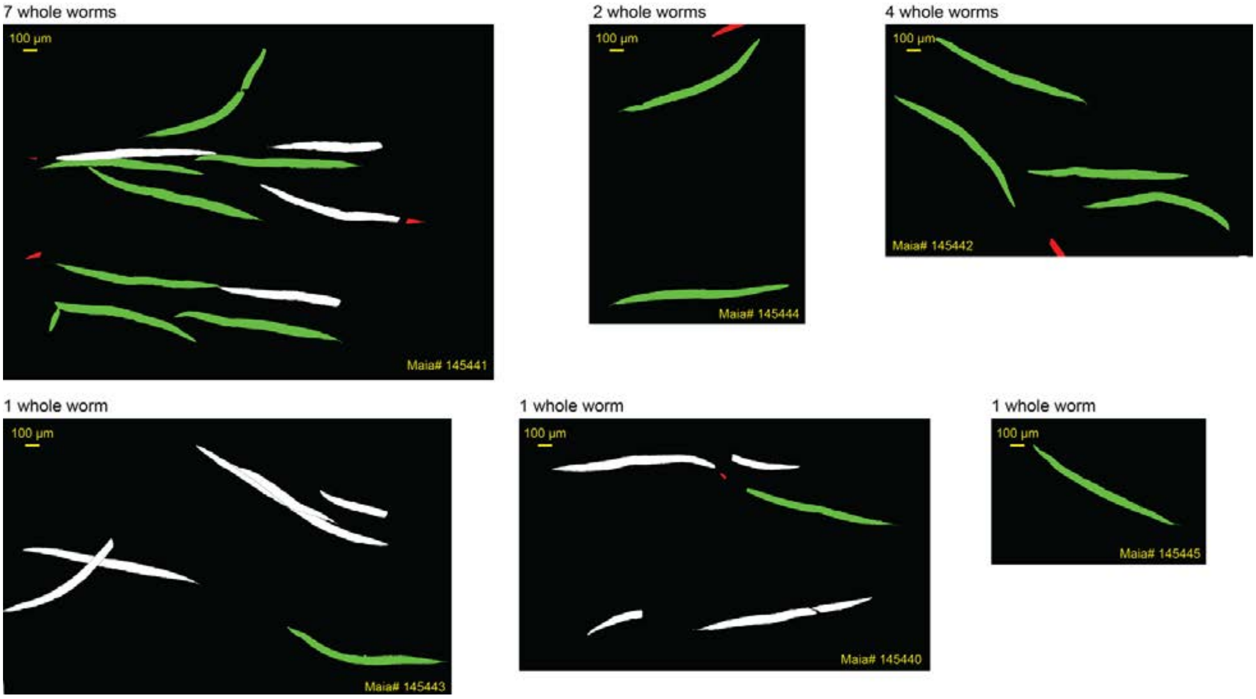
7 whole worms
100 µm
Maia# 145441
2 whole worms
100 µm
Maia# 145444
4 whole worms
100 µm
Maia# 145442
1 whole worm
100 µm
Maia# 145443
1 whole worm
100 µm
Maia# 145440
1 whole worm
100 µm
Maia# 145445

G

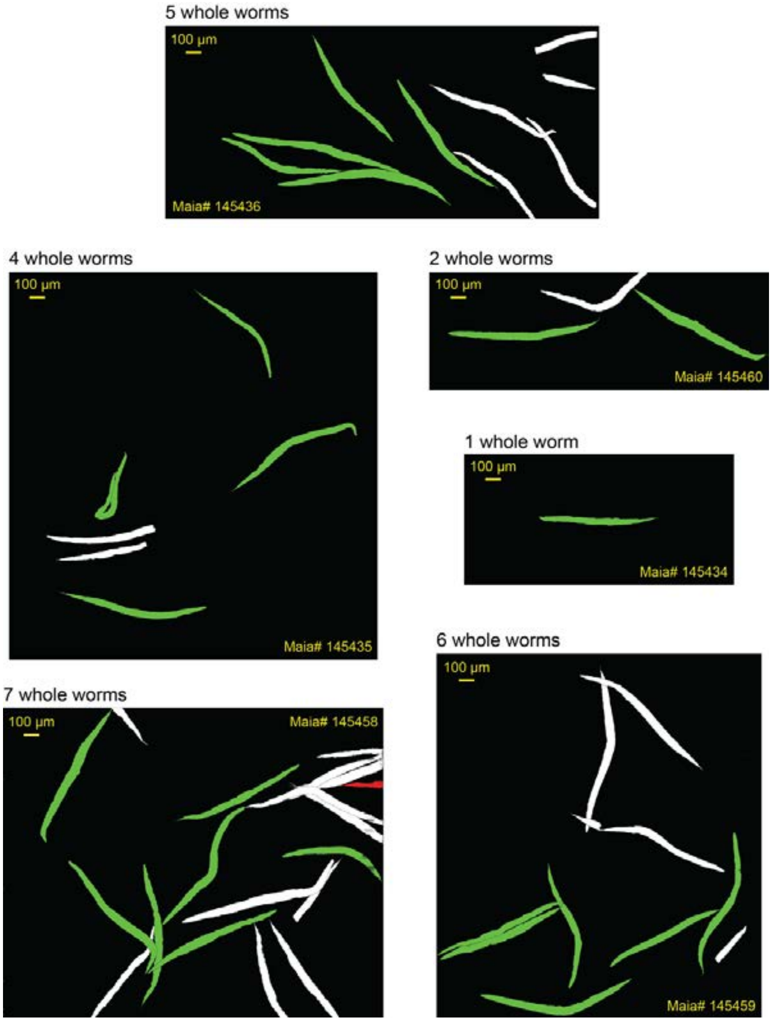
5 whole worms
100 µm
Maia# 145436
4 whole worms
100 µm
Maia# 145435
2 whole worms
100 µm
Maia# 145460
1 whole worm
100 µm
Maia# 145434
7 whole worms
100 µm
Maia# 145458
6 whole worms
100 µm
Maia# 145459
